# Protective Effects Induced by a Hydroalcoholic *Allium sativum* Extract in Isolated Mouse Heart

**DOI:** 10.3390/nu13072332

**Published:** 2021-07-08

**Authors:** Lucia Recinella, Annalisa Chiavaroli, Fabrizio Masciulli, Caterina Fraschetti, Antonello Filippi, Stefania Cesa, Francesco Cairone, Era Gorica, Marinella De Leo, Alessandra Braca, Alma Martelli, Vincenzo Calderone, Giustino Orlando, Claudio Ferrante, Luigi Menghini, Simonetta Cristina Di Simone, Serena Veschi, Alessandro Cama, Luigi Brunetti, Sheila Leone

**Affiliations:** 1 Department of Pharmacy, G. d’Annunzio University of Chieti-Pescara, 66013 Chieti, Italy; lucia.recinella@unich.it (L.R.); annalisa.chiavaroli@unich.it (A.C.); fabriziomasciulli92@gmail.com (F.M.); giustino.orlando@unich.it (G.O.); claudio.ferrante@unich.it (C.F.); luigi.menghini@unich.it (L.M.); disimonesimonetta@gmail.com (S.C.D.S.); veschi@unich.it (S.V.); alessandro.cama@unich.it (A.C.); sheila.leone@unich.it (S.L.); 2 Department of Drug Chemistry and Technology, Sapienza University of Rome, 00185 Rome, Italy; caterina.fraschetti@uniroma1.it (C.F.); antonello.filippi@uniroma1.it (A.F.); stefania.cesa@uniroma1.it (S.C.); francesco.cairone@uniroma1.it (F.C.); 3 Department of Pharmacy, University of Pisa, 56126 Pisa, Italy; era.gorica@phd.unipi.it (E.G.); marinella.deleo@unipi.it (M.D.L.); alessandra.braca@unipi.it (A.B.); alma.martelli@unipi.it (A.M.); vincenzo.calderone@unipi.it (V.C.); 4 Interdepartmental Research Center “Nutrafood: Nutraceutica e Alimentazione per la Salute”, University of Pisa, 56126 Pisa, Italy; 5 CISUP, Centre for Instrumentation Sharing of Pisa University, 56126 Pisa, Italy; 6 Interdepartmental Research Center “Biology and Pathology of Ageing”, University of Pisa, 56126 Pisa, Italy

**Keywords:** garlic, multimethodological evaluation, CIEL*a*b*, HS-SPME/GC–MS, oxidative stress, inflammation, bioinformatics

## Abstract

The aim of the present study was to investigate the possible protective effects of a garlic hydroalcoholic extract on the burden of oxidative stress and inflammation occurring on mouse heart specimens exposed to *E. coli* lipopolysaccharide (LPS), which is a well-established inflammatory stimulus. Headspace solid-phase microextraction combined with the gas chromatography–mass spectrometry (HS-SPME/GC–MS) technique was applied to determine the volatile fraction of the garlic powder, and the HS-SPME conditions were optimized for each of the most representative classes of compounds. CIEL*a*b* colorimetric analyses were performed on the powder sample at the time of delivery, after four and after eight months of storage at room temperature in the dark, to evaluate the color changing. Freshly prepared hydroalcoholic extract was also evaluated in its color character. Furthermore, the hydroalcoholic extract was analyzed through GC–MS. The extract was found to be able to significantly inhibit LPS-induced prostaglandin (PG) E_2_ and 8-iso-PGF_2α_ levels, as well as mRNA levels of cyclooxygenase (COX)-2, interleukin (IL)-6, and nuclear factor-kB (NF-kB), in heart specimens. Concluding, our findings showed that the garlic hydroalcoholic extract exhibited cardioprotective effects on multiple inflammatory and oxidative stress pathways.

## 1. Introduction

Garlic (*Allium sativum* L.) is a member of the Amaryllidaceae family, which represents one of the most widely produced plants all over the world [1]. A wide body of evidence has suggested the multiple biological properties of garlic, which include antioxidant, anti-inflammatory, cardiovascular protective, immunomodulatory, antidiabetic, anti-obesity, and anti-carcinogenic effects [2,3,4,5,6,7]. In particular, an inverse correlation was reported between the consumption of garlic and the progression of cardiovascular disease (CVD). Daily garlic supplementation was found to reduce the serum total cholesterol, inhibit platelet aggregation, decrease blood pressure, and improve vascular function [8,9]. However, the cardioprotective activities of garlic were hypothesized to be strongly dependent on the method of preparation and the availability of the bioactive components in the blood [10,11]. Garlic’s protection against cardiovascular diseases has been hypothesized to be related to its antioxidant [12] and anti-inflammatory [13] activities. Garlic contains a number of bioactive compounds, including polyphenols, flavonoids, flavanols, tannins [14], saponins [15], polysaccharides [16], and sulfur-containing compounds, such as diallyl thiosulfonate (allicin), diallyl sulfide (DAS), diallyl disulfide (DADS), diallyl trisulfide (DATS), *E*/*Z*-ajoene, S-allyl cysteine (SAC) and S-allyl cysteine sulfoxide (alliin) [17]. In particular, allicin, DADS, and DATS appeared to be the most important compounds displaying antioxidant effects [18], and they have been hypothesized to play a key role in cardioprotection [11]. In the present study, we tested the possible beneficial effects of an hydroalcoholic extract of a local variety of garlic (*Allium Sativum* L. var. Nubia-Paceco; Nubia red garlic), which is well known for its strong smell and taste. This variant is a protected denomination of origin product (DOP), which is appreciated all over the world.

Due to the limited distribution of this cultivation in Southern Italy, and the lack of studies on their nutrient contents, we reported here in, to the best of our knowledge, the first potential beneficial effects of *Allium Sativum* L. var. Nubia-Paceco.

In order to define its nutraceutical effect on health and its possible treatment uses, we investigated the possible protective effects of a garlic hydroalcoholic extract on the burden of oxidative stress and inflammation occurring on mouse heart specimens treated with *Escherichia coli* lipopolysaccharide (LPS), which represents a well-known inflammatory stimulus. To this regard, we evaluated the levels of selected biomarkers of oxidative stress and inflammation, including prostaglandin (PG) E_2_, 8-iso-PGF_2α_, as well as cyclooxygenase (COX)-2, tumor necrosis factor (TNF)-α, interleukin (IL)-6, and nuclear factor-kB (NF-kB) mRNA levels. The composition of garlic powder and hydroalcoholic extract was also analyzed by colorimetric and gas chromatography–mass spectrometry analysis (GC–MS). The VOC fraction of garlic powder was notably determined by combining GC–MS analysis with headspace solid-phase microextraction (HS-SPME), wherein several parameters have been tuned to assess the best analytical conditions for each of the most characteristic classes of compounds. Moreover, the hydroalcoholic extract was studied for the quantification of polyphenolic content, by chromatographic high-performance liquid chromatography–diode array (HPLC–DAD) analytical method. Finally, based on the results of the HPLC–DAD analysis, a bioinformatics study was conducted, with the aim to unravel the putative mechanisms underlying the observed pharmacological effects. Overall, our results could provide new insights into the use of this variety, not only as foods, but also as functional and nutraceutical supplements.

## 2. Materials and Methods

### 2.1. Preparation of Garlic Extract

Garlic cloves were kindly provided as powdered and dried material by il Grappolo S.r.l. (Soliera, Modena, Italy). Powder was analyzed by colorimetric and headspace solid-phase microextraction gas chromatography–mass spectrometry analysis (HS-SPME/GC–MS).

One gram plant t^0^ sample was mixed with a solution of ethanol–water (20:80, *v*/*v*) at a final concentration of 1 g/mL. After having been shaken 10 times, the solution was stood for 10 min at room temperature and then centrifuged twice at 3213 RCF for 5 min at 4 °C [19,20]. The supernatant was filtered and then dried (freeze-drying). The dry residue, a yellow sugary solid, with a yield of 2%, was stored at 4 °C, until chemical analyses were performed.

### 2.2. Color Analysis

The garlic powder sample and the hydroalcoholic extract were submitted to colorimetric analysis and analyzed for their color character with a colorimeter X-Rite MetaVue^TM^, equipped with full-spectrum LED illuminant and an observer angle of 45°/0° imaging spectrophotometer. Cylindrical coordinates C*_ab_ and h_ab_ were calculated from a* and b* as known by literature [21]. The same garlic powder sample analysis was performed at the time of delivery (t°), after 4 months (t^4m^) and after 8 months (t^8m^) of storage in the darkness at room temperature (25 ± 2 °C).

### 2.3. GC–MS Analysis of VOC Fraction

#### 2.3.1. HS-SPME/GC–MS of Garlic Powder

Vials (6 mL) have been loaded with 0.1 g of garlic powder without any previous pretreatment. The fiber selection was performed by comparing four different stationary phases, namely, PDMS–DVB, CAR–PDMS, CAR–DVB–PDMS and PA. The following three parameters were tuned to optimize the extraction conditions during the equilibration (eq) and sampling (sa) steps (Table 1): thermostat bath temperature (adjusted at 50, 70, 80, 90 °C) during both the equilibration and sampling steps, equilibration time (t_eq_: 20, 40 min) and sampling time (t_sa_: 20, 35 min). After sampling, fiber was exposed into the GC inlet at 260 °C for 0.5 min. Furthermore, the powder shelf-life was evaluated by analyzing the sample after four-month storage at room temperature. The employed fibers were purchased from Merck Life Science S.R.L. (Milan, Italy). All the analyses were performed in triplicate using an Agilent Technologies 6850 gas chromatograph combined with an Agilent Technologies 5975 mass spectrometer. The following chromatographic conditions were employed: capillary column, HP-5MS (30 m × 0.25 mm inner diameter, film thickness 0.25 µm); inlet temperature, 260 °C; injection mode, split (split ratio 10/1); injection time, 0.5 min; carrier gas, helium (99.995% purity) with a 1.0 mL/min flow; temperature programming, oven was kept at 40 °C for 5 min, then increased by 5 °C/min up to 200 °C, and maintained at this final temperature for 60 min. The mass spectrometer operating values were set as follows: EI energy, 70 eV; source and quadrupole temperatures, 230 °C and 150 °C, respectively; mass scan range, 50–350 *m*/*z*.

#### 2.3.2. GC–MS of Garlic Hydroalcoholic Extract

Garlic hydroalcoholic extract has been redissolved in methanol prior to the injection into the GC–MS device. All the analyses were performed in triplicate and the GC–MS parameters were set as described in Section 2.3.1, with the only exception of the split ratio set to the 40/1 value.

The identification of the volatile compounds was carried out by taking advantage of both mass spectrometry and gas chromatography data. First of all, the experimental EI spectra were compared with those collected in both commercial (FFNSC 3) and free databases (NIST 11, Flavor2). Kovats index (KI) was used as a second parameter to confirm the MS-based identification of the analytes. Misuration of KIs was performed by using a mixture of *n*-alkanes (C7–C40) in the same chromatographic setup, and then compared with values reported in the FFNSC 3 and NIST 11 databases. Chromatographic peaks with a S/N ratio above 3 have been manually integrated without any further modification.

### 2.4. HPLC–DAD Analyses

Garlic hydroalcoholic extract was weighed, dissolved in water and filtered before injection into a HPLC Perkin Elmer apparatus consisting of a Series 200 LC pump, a Series 200 DAD and a Series 200 autosampler, including TotalChrom Perkin Elmer software for the acquisition of data.

Chromatography was performed on an RP phenyl-hexyl column using a mobile phase consisting of acetonitrile and water acidified by 5% acetic acid, with a linear gradient from 98% to 50% aqueous phase in 33′, followed by a second step in isocratic mode, at a flow of 1 mL/min. Calibration curves of alliin (y = 8.96x + 41.84; R^2^ = 0.9974), gallic acid (y = 41.24x + 67.40; R^2^ = 0.9969), protocatechuic acid (y = 20.38x + 49.44; R^2^ = 0.9998) and quercetin-3-galactoside (y = 49.689x + 34.98; R^2^ = 0.9999) were used for the quantitative analyses. All the standards compounds were purchased from Merck Life Science S.R.L. (Milan, Italy).

### 2.5. Toxicological and Pharmacological Studies

#### 2.5.1. Cell Cultures

H9c2 cells (rat cardiomyoblasts, ATTC, Manassas, VA, USA) were maintained in DMEM (Sigma-Aldrich, St. Louis, MO, USA) supplemented with 10% FBS, 1% of 100 unit/mL penicillin and 100 mg/mL streptomycin (Sigma-Aldrich, St. Louis, MO, USA) in T75 red cap tissue culture flasks, at 37 °C in a humidified atmosphere of 5% CO_2_.

#### 2.5.2. Assessment of Cell Toxicity by Garlic Hydroalcoholic Extract in 24 h and 48 h

H9c2 cells that had grown to 90% confluence were plated into a 96-well cell culture transparent plate at a density of 8 × 10^3^ cells per well. After 24 h of cell attachment, the medium was replaced by fresh culture medium and cells were treated with garlic hydroalcoholic extract (1, 10, 50, and 100 μg/mL) or vehicle (culture medium). After 24 or 48 h of incubation, MTT assay was performed, removing all the previous treatment and adding a solution of MTT at a final concentration of 0.5 μg/mL in each well, as previously described [22]. The plate was incubated for 1.5 h at 37 °C in a humidified atmosphere of 5% CO_2_. After that, solubilizing solution was added and the cells were incubated in dark and room temperature in a plate shaker at 200 rpm for 20 min. After 20 min of agitation the absorbance was measured at λ = 570 nm by a multiplate reader (Enspire, Perkin-Elmer, Waltham, MA, USA).

#### 2.5.3. Evaluation of Cell Viability Preservation by Garlic Hydroalcoholic Extract against H_2_O_2_-Induced Cell Damage

H9c2 cells that had grown to 90% confluence were plated into a 96-well cell culture transparent plate at a density of 8 × 10^3^ cells per well. After 24 h of cell attachment, the medium was replaced by fresh culture medium and cells were treated with garlic hydroalcoholic extract (1, 10, 50, and 100 μg/mL) or vehicle (culture medium). After 24 or 48 h incubation, without removing the culture medium with previous treatments, the plate was incubated for other 2 h by adding a just prepared H_2_O_2_ solution (200 μM). At the end of the incubation, MTT assay was performed, removing all the previous treatments and adding a solution of MTT at a final concentration of 0.5 μg/mL in each well. The plate was incubated for 1.5 h at 37 °C in a humidified atmosphere of 5% CO_2_. After that, solubilizing solution was added and the cells were incubated in dark and room temperature in a plate shaker at 200 rpm for 20 min. After 20 min of agitation, measurement of the absorbance was performed at λ = 570 nm by using a multiplate reader (Enspire, Perkin-Elmer, Waltham, MA, USA).

#### 2.5.4. Ex Vivo Studies

Adult C57/BL6 male mice (3-month-old, weight 20–25 g, n = 48) were housed in Plexiglas cages (2–4 animals per cage; 55 cm × 33 cm × 19 cm) and maintained under standard laboratory conditions (21 ± 2 °C; 55 ± 5% humidity) on a 14/10 h light/dark cycle, with ad libitum access to water and food. Housing conditions and experimentation procedures were strictly in agreement with the European Community ethical regulations (EU Directive no. 26/2014) on the care of animals for scientific research. In agreement with the recognized principles of “Replacement, Refinement and Reduction in Animals in Research”, heart specimens were obtained as residual material from vehicle-treated mice randomized in our previous experiments, approved by local ethical committee (‘G. d’Annunzio’ University, Chieti, Italy) and Italian Health Ministry (Project no. 885/2018-PR).

Mice were sacrificed by CO_2_ inhalation (100% CO_2_ at a flow rate of 20% of the chamber volume per min) for heart specimens collection. Heart specimens were then maintained in a humidified incubator with 5% CO_2_ at 37 °C for 4 h (incubation period), in RPMI buffer with added bacterial LPS (10 μg/mL), as previously described [23,24]. During the incubation period, the tissues were challenged with scalar concentrations of garlic hydroalcoholic extract (1–100 μg/mL). After collection of tissue supernatants, measurement of prostaglandin (PG) E_2_ and 8-iso-PGF_2α_ levels (pg/mg wet tissue) was performed by radioimmunoassay (RIA), as previously reported [25]. Specific anti-PGE_2_ and anti-8-iso-PGF_2α_ were developed in the rabbit; the cross-reactivity against other prostanoids was <0.3%. One hundred microliters of prostaglandin standard or sample was incubated overnight at 4 °C with the ^3^H-prostaglandin (3000 cpm/tube; NEN) and antibody (final dilution: 1:120,000; kindly provided by the late prof. G. Ciabattoni), in a volume of 1.5 mL of 0.025 M phosphate buffer. Separation of free and antibody-bound prostaglandins was performed by adding 100 μL 5% bovine serum albumin and 100 μL 3% charcoal suspension, followed by centrifuging for 10 min at 4000× *g* at 5 °C and decanting off the supernatants into scintillation fluid (UltimaGold™, Perkin Elmer) for β-emission counting. The detection limit of the assay method was 0.6 pg/mL.

Total RNA was extracted from the heart specimens using TRI reagent (Sigma-Aldrich, St. Louis, MO, USA), according to the manufacturer’s protocol. One microgram of total RNA extracted from each sample in a 20 µL reaction volume was reverse transcribed using high-capacity cDNA reverse transcription kit (Thermo Fisher Scientific Inc., Monza, Italy). Incubation of the reactions was performed in a 2720 thermal cycler (Thermo Fisher Scientific Inc., Monza, Italy), as previously described [26,27,28]. Gene expression of COX-2, IL-6, NF-kB and TNF-α was evaluated by quantitative real-time PCR using TaqMan probe-based chemistry, as previously reported [26,27,28]. PCR primers and TaqMan probes were purchased from Thermo Fisher Scientific Inc. (Assays-on-Demand Gene Expression Products, Mm00478374_m1 for *COX-2* gene, Mm00443258_m1 for *TNF-α* gene, Mm00446190_m1 for *IL-6* gene, Mm00476361_m1 for *NF-kB* gene, Mm00607939_s1 for *β-actin* gene). β-actin was used as the housekeeping gene. The real-time PCR was performed in triplicate for each cDNA sample in relation to each of the selected genes. Elaboration of the data was performed by using the Sequence Detection System (SDS) software version 2.3 (Thermo Fisher Scientific Inc., Monza, Italy). Relative quantification of gene expression was performed by the comparative 2^−∆∆*C*t^ method [29].

### 2.6. Bioinformatics

Chemical structures preparation and conversion in canonical SMILES was performed by using ChemSketch software. The SMILES were then processed by the Swiss Target Prediction and ADMET Prediction platforms, for prediction of putative targets and pharmacokinetic profile, respectively. Normalization of the name of identified targets was performed according to the UniProt database. Finally, Cytoscape software (3.7.2 version, National Institute of General Medical Sciences (NIGMS), Bethesda, MD, USA) was used to create the components targets illustration network.

### 2.7. Statistical Analysis

At least six different experiments were conducted in triplicate and the data are presented as means ± SEM. Analysis of the data was performed by the software GraphPad Prism 6.0 (Graphpad Software Inc., San Diego, CA, USA) using one-way analysis of variance (ANOVA), followed by Bonferroni’s multiple comparison post hoc test. As for gene expression analysis, 1.00 (calibrator sample) was considered the theoretical mean for the comparison [28]. A *p*-value < 0.05 was considered as the limit of statistically significant differences between mean values. The number of animals randomized for each experimental group was calculated on the basis of the “Resource Equation” N = (E + T)/T (10 ≤ E ≤ 20) [30].

## 3. Results and Discussion

### 3.1. GC–MS Analysis of VOC Fraction

#### 3.1.1. Garlic Powder

**Figure 1:**
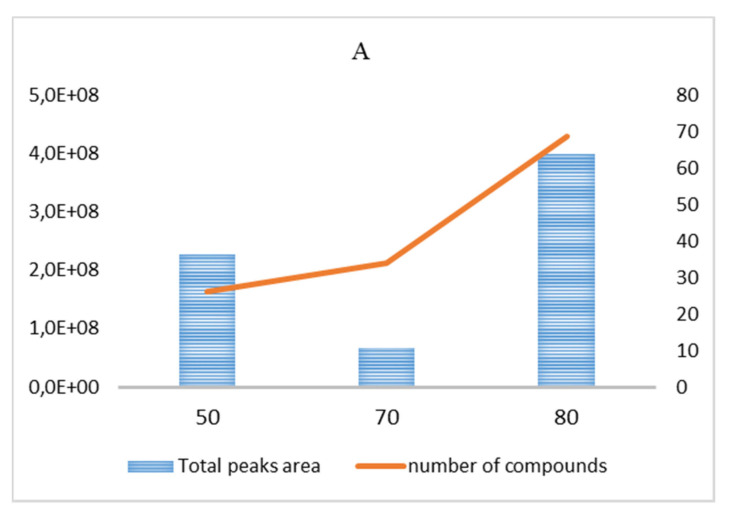

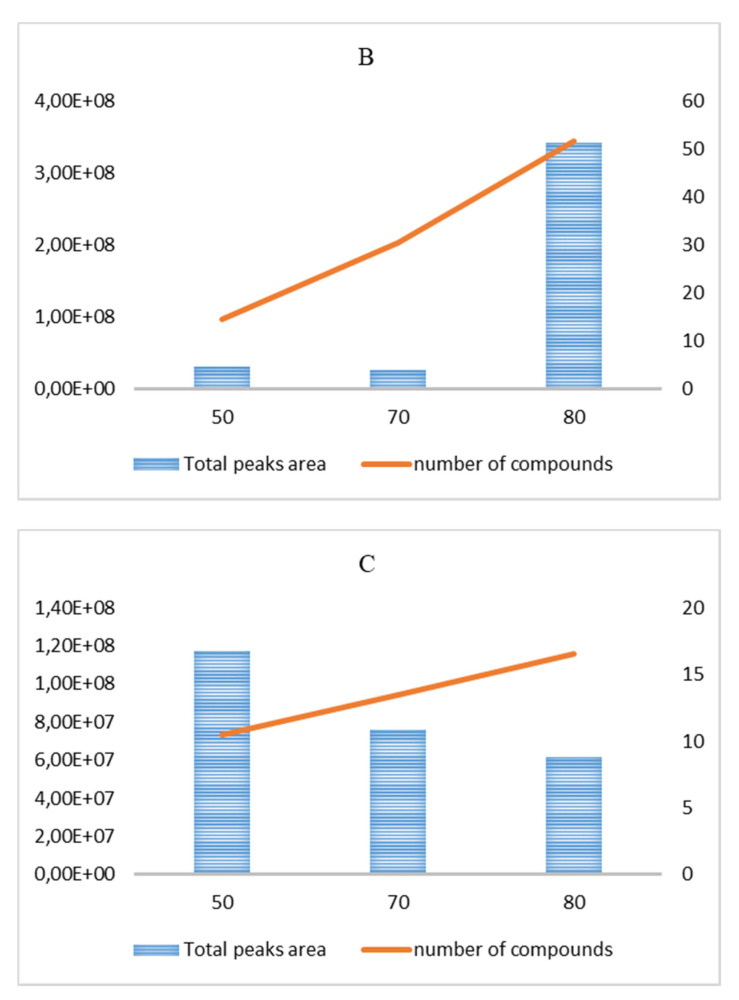
Optimization of HS-SPME conditions using (**A**) DVB–CAR–PDMS, (**B**) PDMS–DVB, (**C**) CAR–PDMS fibers with the following settings: t_eq_ = 20 min, t_sa_ = 15 min. Total peaks area (primary axes) and number of detected peaks (secondary axes) are plotted vs. equilibration/sampling temperature (in °C).

The presence of specific compounds in garlic samples was found to be mainly related to the mode of garlic preparation and extraction [31]. SPME fibers have been compared through the extraction efficiency, which is well represented by the total chromatographic areas obtained for t_eq_ = 20 min and t_sa_ = 15 min in the 50–80 °C temperature range (Figure 1A–C; in Figure S1, the results obtained for t_eq_ = 20 min and t_sa_ = 35 min in the same temperature range are reported). The primary and secondary axes (Figure 1A–C and Figure S1A–C) indicate the total chromatographic area and the number of chromatographic peaks, respectively. The significant sample darkening attained at t_eq_ = 90 °C, and the independence from t_eq_ in the 20–40 min range, suggested us to focus the investigation to the 50–80 °C range with t_eq_ = 20 min. Finally, the polar PA cartridge is no longer considered in the following discussion, due to the very poor extraction efficiency (the results are not reported).

**Figure 2:**
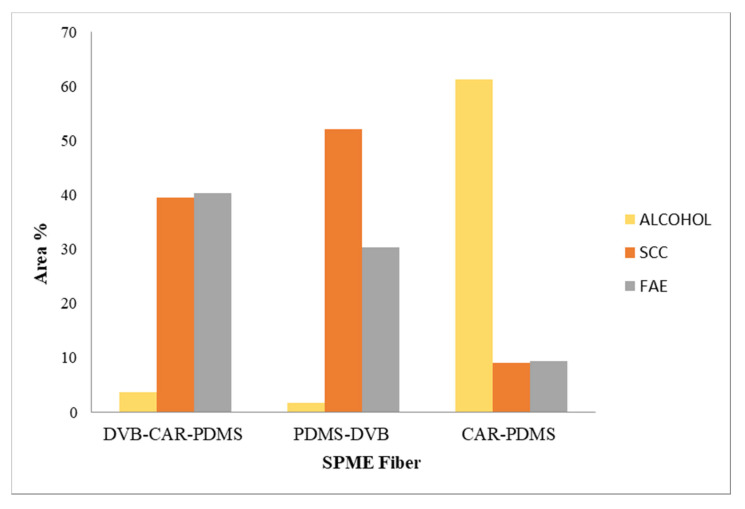
Distribution of the principal classes of volatile compounds detected (HS-SPME conditions: T = 80 °C; t_eq_ = 20 min, t_sa_ = 15 min).

The best efficiency, in parallel to the higher number of detected compounds, was obtained with the DVB–CAR–PDMS fiber (T_eq/sa_ = 80 °C, t_eq_ = 20 min and t_sa_ = 15 min; Figure 1A), which extracted three prevailing classes of compounds (Table 2), namely, alcohols, sulfur-containing compounds (SCC), and fatty acid esters (FAE), whose relative abundance crucially changes with the HS-SPME conditions (Figure 2 and Figure S2). The fiber selectivity presents remarkable differences; the DVB–CAR–PDMS cartridge has a similar selectivity for FAE as well as for SCC (39.4% and 40.3%, respectively), and a very low affinity for alcohols (3.6%). This pattern is reversed when the CAR–PDMS results are considered; indeed, alcohols definitely represent the principal class of compounds absorbed by this phase (61.2%). The selectivity of the PDMS–DVB cartridge is pronouncedly oriented toward SCC (52.1%), and significantly less toward FAE (30.4%). In the most efficient extractions performed in a different set of HS-SPME parameters (t_eq_ = 20 min and t_sa_ = 35 min), PDMS–DVB and CAR–PDMS fibers show a similar pattern to the result obtained for t_sa_ = 15 min (Figure 2 and Figure S2). On the contrary, the DVB–CAR–PDMS one extracted FAE very efficiently (69.1% in Figure S2).

In the optimized conditions (Table 2), diallyl trisulfide is the most abundant component (22.0%), followed by ethyl linoleate (17.8%), ethyl palmitate (9.68%), diallyl tetrasulfide (6.80%), and diallyl disulfide (6.18%). Diallyl trisulfide is also the major analyte extracted by the PDMS–DVB fiber in the same analytical conditions (37.52% in Table S1). The alcohol-selective CAR–PDMS phase extracts allyl alcohol very efficiently (31.04%), probably arising from alliin degradation [32], as well as 2,3-butanediol (28.06%). It is noteworthy that the VOC distribution was not altered by the four-month room temperature storage (Table S2).

#### 3.1.2. Garlic Hydroalcoholic Extract

The analysis of the hydroalcoholic extract basically revealed the exclusive presence of the following two classes of compounds: fatty acid esters (FAE: 22.16%) and long-chain n-alkanes (LA: 53.47%), besides a certain number of unknown compounds (Table 3). The most representative FAE are methyl and ethyl linoleate (6.87 and 4.76%, respectively).

### 3.2. Colorimetric Analysis

**Figure 3:**
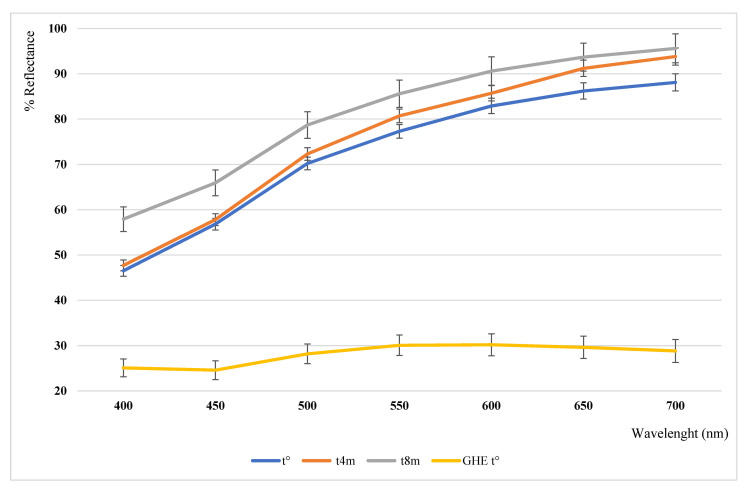
Reflectance curves of the analyzed garlic samples.

The color parameters and relative reflectance curves of the analyzed samples are reported in Table 4 and Figure 3.

The garlic powder showed a very bright (L* = 90.15) yellow color (b* = 16.02), which remains unvaried for the first four months of storage, at room temperature and in the darkness (t^4m^, ΔE = 1.11 in respect to the limit detectable for the human eye, recognized as ΔE = 1.00). After another four months of storage, at t^8m^, the color has undergone a slight discoloration (b* = 13.78 vs. 16.02 at t°), which could account for a slight carotenoid and/or polyphenol degradation. The presence of a little carotenoid content in *Allium* spp. was previously reported [33], and we also reported the carotenoid bleaching in a shelf-life study on powdered infant milk formulas [34]. In regards to polyphenol compounds, these could contribute to an overall pale yellow color, as also reported in our previous study of crop and wild *Allium* spp. [35]. These compounds also justify a color changing over time, due to their molecular structures and lability, as confirmed by the HPLC–DAD analysis (discussed below), which denotes the presence of flavonoid derivatives as main compounds. A paper reported flavonoid autoxidation as a cause of food instability [36], especially for food containing high levels of iron and copper ions, as garlic does, which favor the autoxidation processes.

The analysis of the hydroalcoholic extract is only in part comparable to the previously obtained results [35], denoting a lesser yellow character (b* = 6.81 vs. 11–25 as previously found). Anyway, different parameters should be considered, since the expressed color could be deeply influenced by the different adopted solvent ratio and the correlated sample opacity, due to the presence of components that are only partially solubilized.

### 3.3. HPLC–DAD Analysis

**Figure 4:**
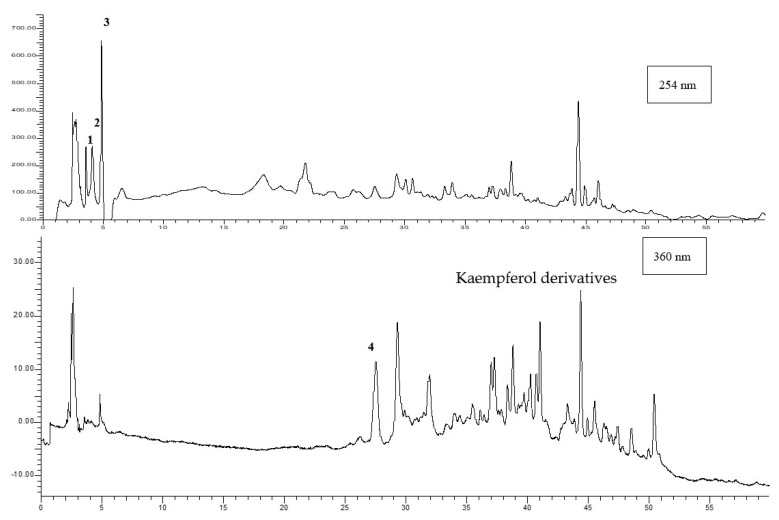
Example chromatograms of garlic hydroalcoholic extract.

An example chromatogram of the garlic hydroalcoholic extract is reported in Figure 4.

From the chromatographic profiles recorded at 254 and 360 nm, it is possible to identify rich phenolic acid and flavonoid contents, which are characterized by the presence of alliin (1), gallic acid (2), protocatechuic acid (3), some flavonoids (T_r_ 18–22 min), and other flavonoids, such as quercetin-3-D-galactoside (4), followed by another ten flavonoid peaks (among which are probably kaempferol derivatives, as reported in the literature [37,38,39].

Alliin (1.2 mg/g dry extract), gallic acid (2.3 mg/g dry extract), protocatechuic acid (11.7 mg/g dry extract), and quercetin-3-galactoside (0.3 mg/g dry extract) were quantified on the basis of the calibration curves, as well as the other most relevant peaks revealed at 360 nm, which were quantified as the sum and expressed as quercetin-3-galactoside (2.5 mg/g dry extract). The results are only in part comparable to those reported in the literature, from which chromatograms are not always available. Anyway, as known, each cultivar expresses a peculiar phytocomplex and different analyte contents. Alliin, whose content is deeply influenced by the agronomic conditions, nitrogen and sulfur supplementation, was previously reported in the range of 0.5–33.4 mg/g [40], as well as a high variability of composition and content being reported in regards to the phenolic acids and flavonoids class [41].

### 3.4. Bioinformatics

Considering the results of the quantitative HPLC–DAD analysis, a bioinformatics study was conducted on the prominent compounds identified and quantified in the extract, namely, gallic acid, protocatechuic acid, alliin, and quercetin-3-galactoside, in order to predict the pharmacokinetics and putative targets. Pharmacokinetics parameters, such as gastrointestinal absorption and bioavailability, were evaluated through the ADMET Prediction platform; whereas the SwissTargetPrediction software was employed for unravelling the putative interactions of gallic acid, protocatechuic acid, alliin, and quercetin-3-galactoside with human proteins. Intriguingly, all of the selected phytocompounds were predicted to be adsorbed at the gastrointestinal level, with bioavailability ranging from 20 to 30% for an orally administered dose. Additionally, the present phytochemicals showed no putative interactions with p-glycoprotein and P450 cytochromes, thus further suggesting the rationale for the in vivo administration of the tested extract. Regarding the putative mechanisms of action, the components–targets analysis conducted pointed to interactions with different families of proteins involved in the inflammatory response, including secreted proteins and oxidoreductases, among which are cytokines and COX-2, respectively. 

### 3.5. Toxicological and Pharmacological Studies

**Figure 5:**
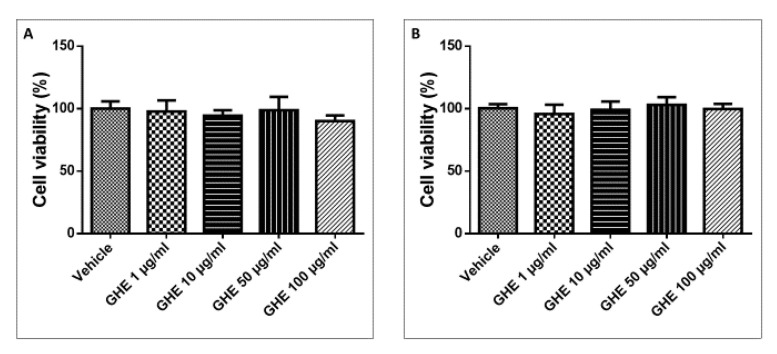
MTT assay of H9c2 cells exposed to garlic hydroalcoholic extract (GHE) (1, 10, 50, and 100 μg/mL) for 24 h (**A**) and 48 h (**B**), in basal conditions. Data are reported as means ± SEM.

The garlic hydroalcoholic extract (1, 10, 50, and 100 μg/mL) was tested in vitro to evaluate its effects on the viability of H9c2 cells. No significant effect on H9c2 cell proliferation was observed after the addition of the garlic hydroalcoholic extract to the cell medium at various concentrations (1, 10, 50, and 100 μg/mL) for 24 h and 48 h (Figure 5A,B).

**Figure 6:**
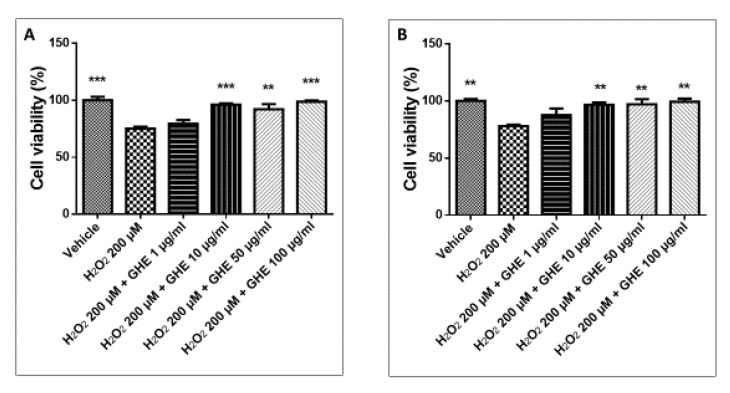
MTT assay of H9c2 cells exposed to garlic hydroalcoholic extract (GHE) (1, 10, 50, and 100 μg/mL) for 24 h (**A**) and 48 h (**B**), and challenged with 200 µM H_2_O_2_. Data are reported as means ± SEM. ANOVA, *p* < 0.01; ** *p* < 0.01, *** *p* < 0.001 vs. H_2_O_2_ group.

Moreover, when the H9c2 cells were treated with H_2_O_2_, their viability was significantly decreased, but the garlic hydroalcoholic extract (10, 50, and 100 μg/mL) was found to be effective in reverting the cytotoxicity at both the experimental times (Figure 6A,B).

**Figure 7:**
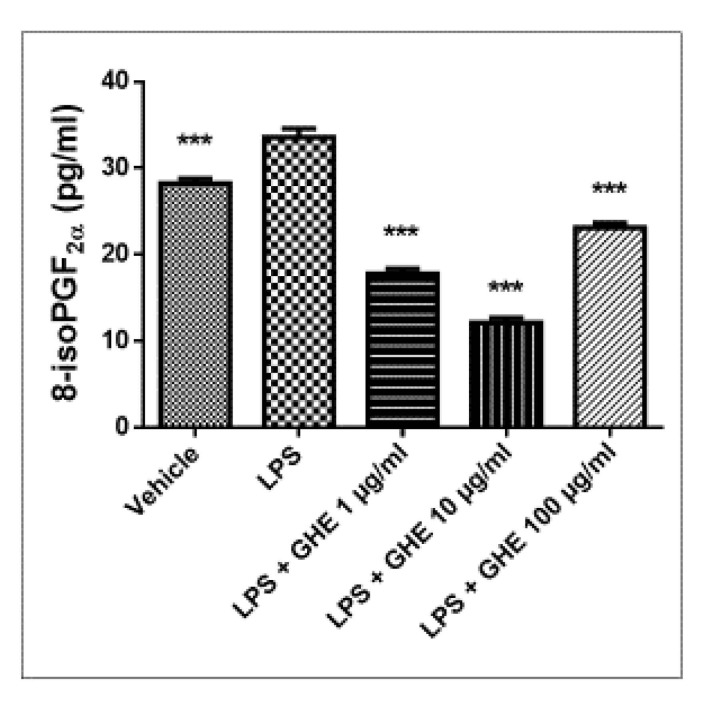
Effects of garlic hydroalcoholic extract (GHE) (1, 10, and 100 μg/mL) on 8-iso-PGF_2α_ levels in mouse heart specimens. Data are reported as means ± SEM. ANOVA, *p* < 0.0001; *** *p* < 0.001 vs. LPS group.

On the basis of these results, a second set of experiments was conducted, with the aim to investigate the protective effects induced by the garlic hydroalcoholic extract (1, 10 and 100 μg/mL) on oxidative stress and inflammatory pathways in mouse heart specimens challenged with LPS. Isolated tissues ex vivo, treated with LPS, have been described as a validated experimental model to determine the modulatory activities of plant-derived extracts and drugs on inflammation and oxidative stress [23,24,42]. Inflammatory processes and oxidative stress are known to play a pathogenic role in a number of chronic diseases, such as cardiovascular diseases, atherosclerosis, and diabetes [43,44]. Cardioprotective and anti-atherosclerotic effects, including an improvement in the blood lipid profile, inhibition of cholesterol biosynthesis, regulation of blood pressure, and inhibition of platelet aggregation, have been reported for garlic-based preparations [45,46]. In particular, the effects of garlic hydroalcoholic extract (1, 10 and 100 μg/mL) were measured against the increased levels of pro-inflammatory and pro-oxidant mediators, such as PGE_2_ and 8-iso-PGF_2α_. The pro-oxidant mediator 8-iso-PGF_2α_ is an isomer of prostaglandins, and is produced by the oxygen radical-catalyzed peroxidation of membrane arachidonic acid, which plays a critical role as a stable marker of lipid peroxidation and oxidative stress [47]. We observed that garlic hydroalcoholic extract (1, 10 and 100 μg/mL) decreased LPS-induced 8-iso-PGF_2α_ in a dose-independent manner, on mouse heart specimens (Figure 7).

Cardioprotective effects, induced by the garlic, were consistently demonstrated on primary cultured cardiac myocytes, fibroblasts and endothelial cells, through the reduction in reactive oxygen species-dependent signaling pathways [48]. In agreement, a garlic hydroalcoholic extract was found to be effective in decreasing the serum oxidative stress index and total oxidative status, nitric oxide and malondialdehyde production, in a rat experimental model of acute inflammation [49]. In addition, garlic extracts exhibited significant antioxidant activity and protective effects against oxidative DNA damage [50], independently of the processing method. The inhibitory effects induced by the garlic hydroalcoholic extract on 8-iso-PGF_2α_ levels could be related to its phenolic acid and flavonoid content, with particular regard to the levels of protocatechuic acid and gallic acid [41,51,52,53,54]. In this context, polyphenol compounds were found to be able to exert cardioprotective effects through the inhibition of oxidative stress and inflammation [55,56,57,58,59]. In particular, it has been demonstrated that protocatechuic acid, one of the main secondary metabolites of garlic, exhibits antioxidant and anti-inflammatory effects [60]. Moreover, it was shown to inhibit the inflammation response, platelet aggregation, and cardiomyocytes apoptosis on myocardial ischemia/reperfusion injury [61]. In addition, both antihypertensive and antioxidant effects of protocatechuic acid have been described in hypertensive rats [62]. Gallic acid has also been hypothesized to protect the heart through the inhibition of lipid peroxidation, due to its scavenging effects on superoxide and hydroxyl radicals [63]. Furthermore, gallic acid pretreatment inhibited the serum levels of cardiac marker enzymes, such as cardiac troponin T, which is probably related to the decrease in myocardial damage [64].

**Figure 8:**
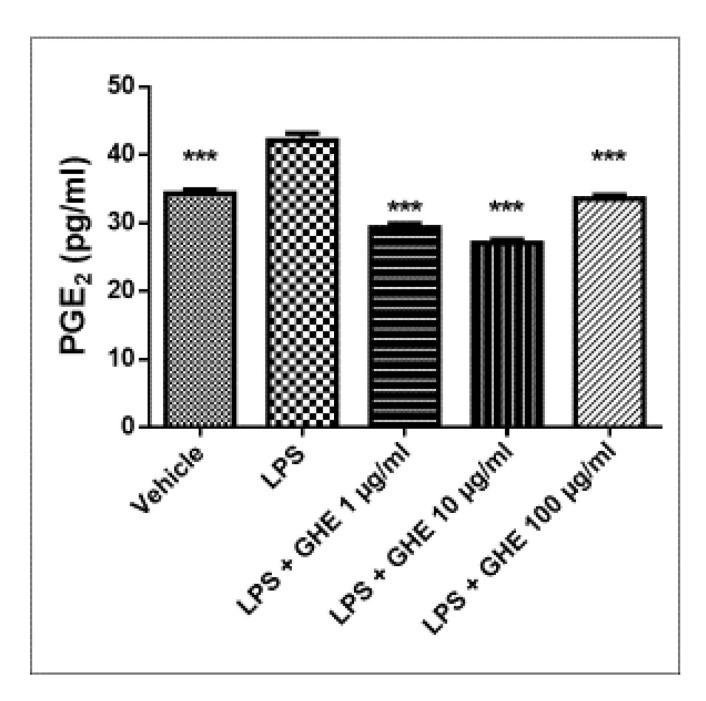
Effects of garlic hydroalcoholic extract (GHE) (1, 10, and 100 μg/mL) on 8-iso-PGE_2_ levels, in mouse heart specimens. Data are reported as means ± SEM. ANOVA, *p* < 0.0001; *** *p* < 0.001 vs. LPS group.

We also showed that garlic hydroalcoholic extract inhibited LPS-induced PGE_2_ levels in heart specimens (Figure 8), supporting its cardioprotective effects.

**Figure 9:**
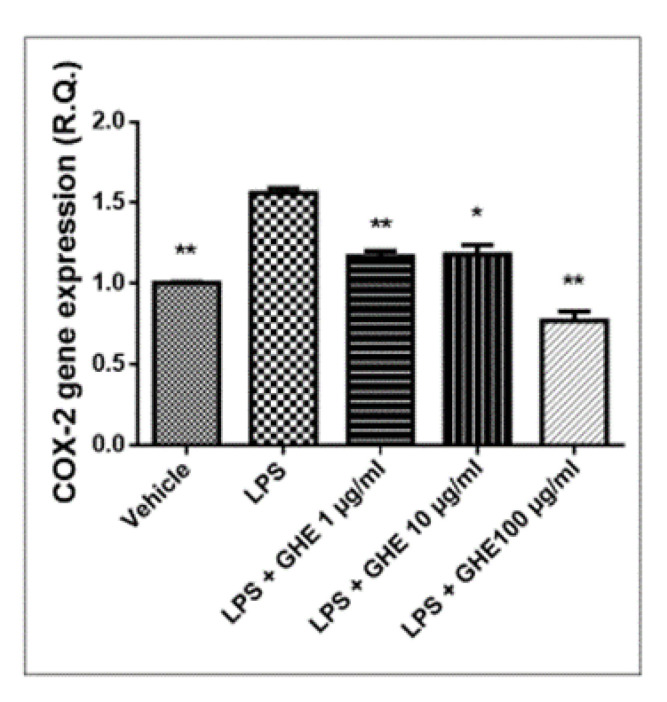
Relative quantification of *COX-2* gene expression in mouse heart specimens treated with garlic hydroalcoholic extract (GHE) (1, 10, and 100 μg/mL), ex vivo. Data are reported as means ± SEM. ANOVA, *p* < 0.05; ** *p* < 0.01; * *p* < 0.05 vs. LPS group.

**Figure 10:**
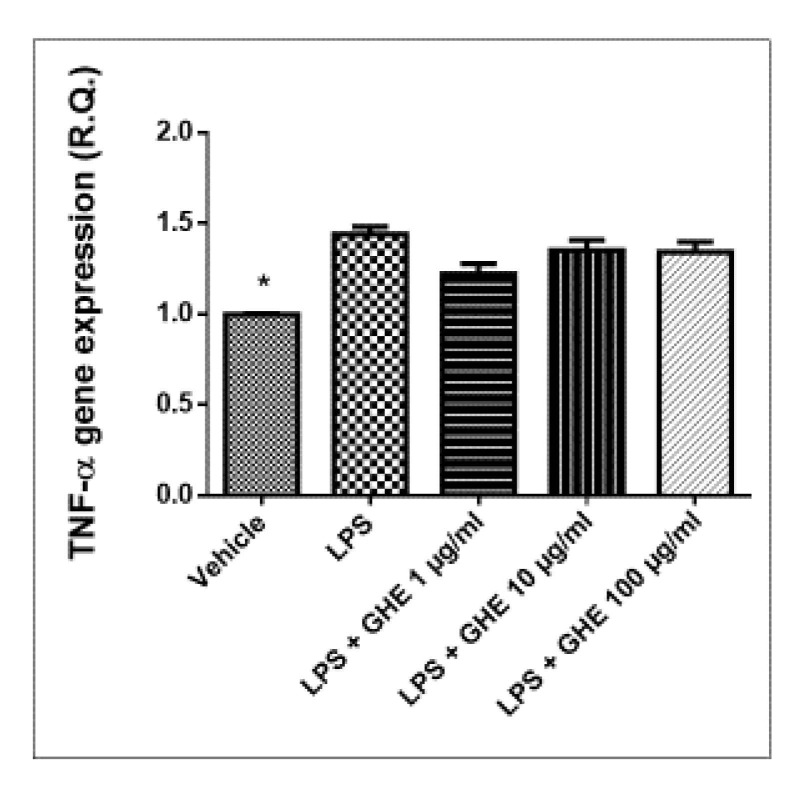
Relative quantification of *TNF-α* gene expression in mouse heart specimens treated with garlic hydroalcoholic extract (GHE) (1, 10, and 100 μg/mL), ex vivo. Data are reported as means ± SEM. ANOVA, *p* < 0.05; * *p* < 0.05 vs. LPS group.

Pro-inflammatory PGE_2_ is produced by COX-2, in both neoplastic and inflamed tissue. The expression of the inducible enzyme COX-2 is known to be stimulated by mitogenic and inflammatory stimuli, such as cytokines and LPS [65]. In turn, LPS stimulated the macrophage production of pro-inflammatory cytokines, such as TNF-α, IL-1β, and IL-6, as well as mediators, including PGE_2_ [66,67]. The mRNA levels of TNF-α, IL-6, NF-κB, and COX-2 were evaluated, as well. In this context, we observed that garlic hydroalcoholic extract (1, 10 and 100 μg/mL) was able to reduce the mRNA levels of COX-2, without affecting *TNF-α* gene expression, in mouse heart specimens challenged with LPS inflammatory stimulus (Figure 9 and Figure 10).

**Figure 11:**
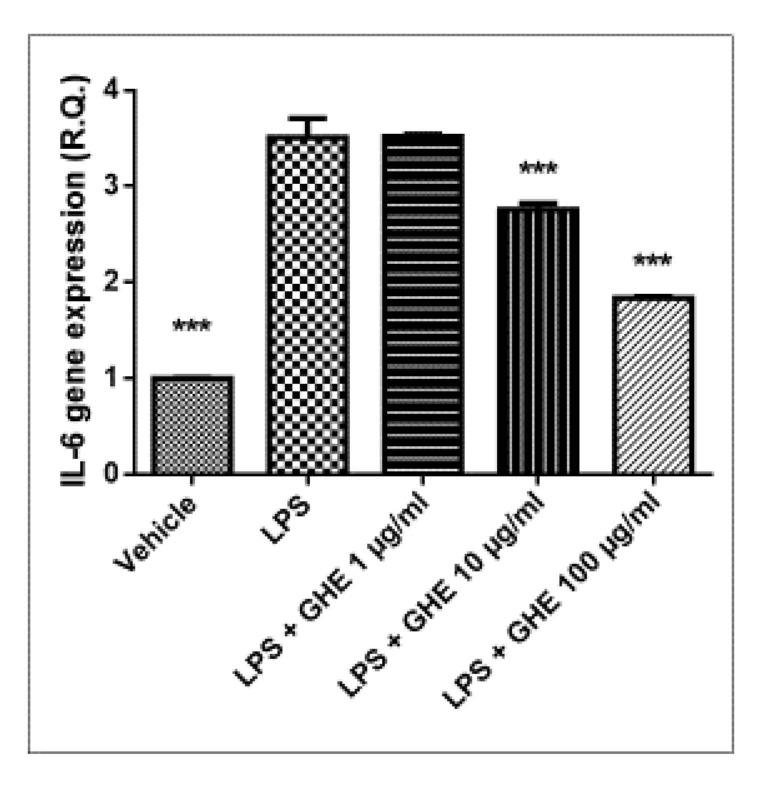
Relative quantification of *IL-6* gene expression in mouse heart specimens treated with garlic hydroalcoholic extract (GHE) (1, 10, and 100 μg/mL), ex vivo. Data are reported as means ± SEM. ANOVA, *p* < 0.01; *** *p* < 0.001 vs. LPS group.

**Figure 12:**
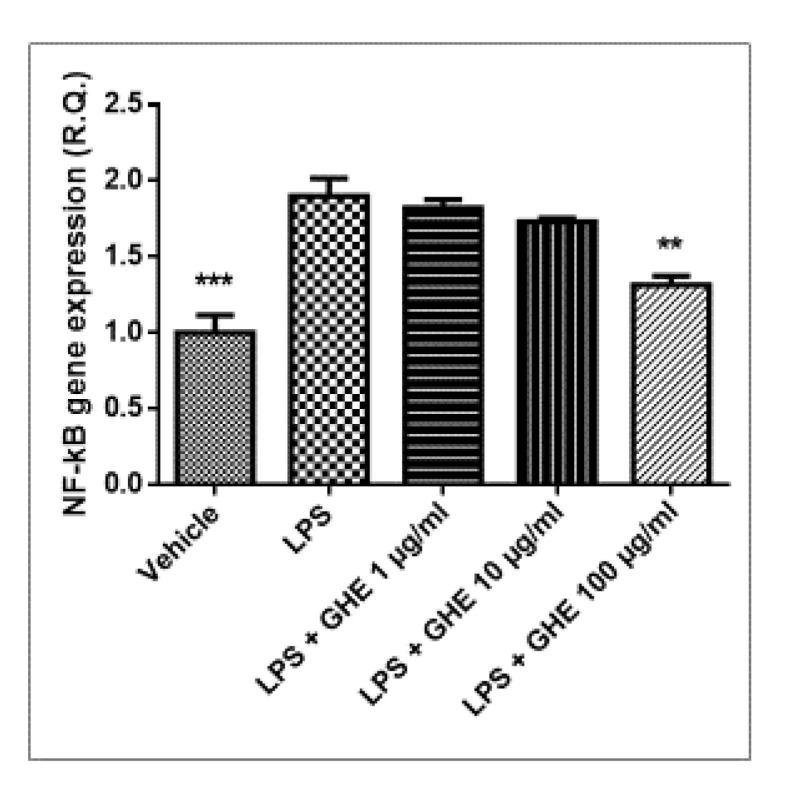
Relative quantification of *NF-kB* gene expression in mouse heart specimens treated with garlic hydroalcoholic extract (GHE) (1, 10, and 100 μg/mL), ex vivo. Data are reported as means ± SEM. ANOVA, *p* < 0.01; ** *p* < 0.001; *** *p* < 0.001 vs. LPS group.

Selective COX-2 inhibition has been reported to improve endothelium-dependent vasodilation, and decrease low-grade chronic inflammation and oxidative stress in coronary artery disease [68]. The higher extract concentrations were effective in counteracting LPS-induced IL-6 mRNA levels (Figure 11), whereas only the highest concentration (100 μg/mL) suppressed NF-kB mRNA induced by LPS treatment in heart specimens (Figure 12).

Accordingly, Hodge et al. (2002) found that garlic extracts (≥10.0 μg/mL) significantly stimulated the synthesis of IL-10, an anti-inflammatory cytokine, in LPS-treated human whole-blood cultures, and suppressed the monocyte production of pro-inflammatory cytokines, including TNF-α, IL-6 and IL-8 [69]. The immunomodulatory effects of garlic were also confirmed by another study [70], which showed that garlic extracts stimulated IL-10 production, while they reduced TNF-α and IL-6 production, in LPS-stimulated human placental explants. These results are in agreement with the anti-inflammatory effects observed in mouse heart specimens. On the other hand, the differences in the modulatory effects of garlic extracts on *TNF-*α gene expression could be related to the different experimental paradigms used. The modulatory effects induced by garlic in human whole blood has also been suggested to be related to a reduction in NF-kB activity [71]. NF-kB is a transcription factor that is strongly involved in the modulation of proinflammatory gene expression, such as COX-2, in response to LPS [72]. Increased activity of NF-kB is also related to inflammatory diseases, including arthritis and atherosclerosis [73]. Thus, we can suggest that the inhibition of *NF-kB* gene expression could be a common inhibitory pathway for the observed COX-2 and PGE_2_ activities, following treatment with garlic extract. In agreement, Park et al. (2014) reported that ethyl linoleate from garlic was able to inhibit COX-2 expression and PGE_2_ production in LPS-activated RAW264.7 cells, by inhibiting NF-kB activation [74]. Actually, we speculate that the anti-inflammatory effects exerted by the garlic hydroalcoholic extract could also be related, at least in part, to the phenol and flavonoid content. In particular, the presence of gallic acid and protocatechuic acid is consistent with the reduction in the tested pro-inflammatory biomarkers [75,76,77]. In agreement, protocatechuic acid supplementation in diet was found to able to prevent coagulation and inflammation in streptozotocin-induced diabetic mice, through a reduction in pro-inflammatory cytokines, including IL-6 and monocyte chemoattractant protein-1 (MCP-1) levels in the heart and kidneys [78]. Moreover, oral administration of gallic acid was able to ameliorate cardiac damage, by inhibiting the myocardial markers of inflammation, including nitric oxide [79]. Considering the limitations of the in vitro and ex vivo experimental paradigms [80,81,82], further studies, including in vivo evaluations, are required to confirm the direct interaction of specific cells with the tested extract. 

## 4. Conclusions

In conclusion, our findings showed that the garlic hydroalcoholic extract exhibited cardioprotective effects on multiple inflammatory and oxidative stress pathways. The chemical profile revealed the presence of selected classes of compounds (alcohols and fatty acid esters) in the hydroalcoholic extract, which can be responsible for such effects. Sulfur-containing compounds, describing the chemical fingerprint of this natural matrix, were revealed only as a powder in the sample. Intriguingly, the prominent phenolic compounds present in the extract, namely, gallic acid, protocatechuic acid, alliin, and quercetin-3-galactoside were also predicted to be adsorbed at the gastrointestinal level, with the putative bioavailability ranging from 20 to 30%. The same phytochemicals were also predicted to interact with cytokines and COX-2; this could explain, albeit partially, the observed inhibitory effects induced by garlic extract on the selected pro-inflammatory biomarkers. The present findings also add to the cardioprotective effects exerted by herbal extracts that are rich in phenolics, and are orally administered [71]; this further supports the potential use of the present garlic extract in contrasting oxidative stress and inflammatory processes occurring during cardiovascular diseases. However, future studies using independent experimental paradigms are required for an accurate evaluation of the in vivo activity.

## Figures and Tables

**Table 1:** Optimized HS-SPME parameters during the equilibration (eq) and sampling (sa) steps.

Temperature (°C)	50	70	80	90
Equilibration time (min)	20			
	40			
Sampling time (min)	15			
	35			

**Table 2:** HS-SPME/GC–MS results obtained using DVB–CAR–PDMS fiber (t_eq_ = 20 min and t_sa_ = 15 min at T_eq/sa_ = 80) °C).

Compound	Class	Area%	RI	RI_L_^a^
Ethanol ^b^	Alcohols	0.10	-	-
Allyl alcohol ^b^	Alcohols	3.07	-	-
Trichloromethane ^b^	Other	0.12	-	-
Hexanal	Other	0.01	809	800
Diallyl sulfide	SCC	0.09	859	857
Methyl allyl disulfide	SCC	0.22	917	919
Dimethyl trisulfide	SCC	0.04	970	969
2-Thiophenecarboxaldehyde	SCC	0.07	1011	1005
Diallyl disulfide	SCC	6.18	1081	1090
Allyl methyl trisulfide	SCC	2.08	1140	1137
2,3-Dihydro-3,5-dihydroxy-6-methyl-4H-pyran-4-one	Other	0.78	1157	1162
3-Vinyl-1,2-dithiacyclohex-5-ene	SCC	0.07	1190	1185
3-Vinyl-1,2-dithiacyclohex-4-ene	SCC	0.24	1216	1214
Diallyl trisulfide (allitridin)	SCC	22.0	1305	1300
Propenyl propyl trisulfide	SCC	0.16	1316	1314
Eugenol	Alcohols	0.54	1366	1357
Methyl 1-propenyl tetrasulfide	SCC	0.93	1369	1368
Allyl 1-methylthio propyl disulfide	SCC	0.85	1386	1387
Diallyl tetrasulfide	SCC	6.80	1548	1539
Tridecyl methyl ketone	Other	0.15	1700	1697
2,4-dimethyl-5,6-dithia-2,7-nonadienal	SCC	3.10	1789	1788
Myristic acid	Other	0.11	1795	1794
FAE (unidentified)	FAE	0.40	1823	-
Phthalate diisobutyl	Other	0.18	1876	1858
Methyl palmitate	FAE	1.26	1926	1925
Phthalate dibutyl	FAE	0.39	1969	1960
Ethyl palmitoleate	FAE	0.70	1972	1975
Ethyl palmitate	FAE	9.68	1995	1993
Propyl palmitate	FAE	0.31	2089	2077
Methyl linoleate	FAE	2.62	2096	2093
Methyl elaidate	FAE	0.56	2101	2107
Ethyl linoleate	FAE	17.8	2161	2164
Ethyl oleate	FAE	4.93	2165	2173
Ethyl stearate	FAE	0.16	2193	2198
Methyl-9-12-heptadecadienoate ^b^	FAE	2.70	2242	-
29 unknown		10.6		
**Class**	**Area%**			
Alcohol	3.7			
SCC	42.8			
FAE	41.5			
Others	1.4			

^a^ RI reported in literature ^b^ MS-only identification method. SCC: sulfur-containing compounds. FAE: fatty acid esters.

**Table 3:** GC–MS analysis of hydroalcoholic garlic extract.

Hydroalcoholic Extract				
**Compound**	**Class**	**Area%**	**RI**	**RI_L_^a^**
Diisobutyl phthalate	Other	0.60	1883	1858
Methyl palmitate	FAE	2.07	1933	1925
Ethyl palmitate	FAE	2.14	2001	1993
Methyl linoleate	FAE	6.87	2103	2093
FAE (unidentified)	FAE	2.69	2107	-
Ethyl linoleate	FAE	4.76	2167	2164
FAE (unidentified)	FAE	3.63	2171	-
Docosane	LA	5.95	2202	2200
Tricosane	LA	11.98	2302	2300
Tetracosane	LA	16.48	2402	2400
Pentacosane	LA	12.75	2502	2500
Hexacosane	LA	6.31	2602	2600
unknown		23.76		
**Class**	**Area%**			
FAE	22.16			
LA	53.47			

^a^ RI values reported in literature.

**Table 4:** Colorimetric CIEL*a*b* parameters of garlic powder and hydroalcoholic extract.

	L*	a*	b*	C*	h°	ΔC*_ab_	Δh_ab_	ΔE
**Powder t°**	90.15	0.47	16.02	16.03	88.32			
**Powder t^4m^**	91.67	0.88	17.31	17.33	87.08	1.30 Brighter	−0.36 More red	1.11
**Powder t^8m^**	93.77	0.45	13.77	13.78	88.13	−2.25 More opaque	−0.05 More red	1.98
**GHE**	61.04	−0.79	6.81	6.86	96.65	−9.17 More opaque	1.52 More green	12.06

GHE = garlic hydroalcoholic extract. Reported value represent the mean of four measurements. Mean error <2%. ΔE represents the overall color variation, using powder at t° as reference, ΔE = [(L*_2_ − L*_1_)^2^ + (a*_2_ − a*_1_)^2^ + (b*_2_ − b*_1_)^2^]^1/2^.

## Data Availability

The data presented in this study are available on request from the corresponding author.

## Supplementary Materials

The following are available online at https://www.mdpi.com/article/10.3390/nu13072332/s1. Figure S1: Optimization of HS-SPME conditions using (A) DVB–CAR–PDMS, (B) PDMS–DVB, (C) CAR–PDMS fibers with the following settings: t_eq_ = 20 min, t_sa_ = 35 min. Total peaks area (primary axes) and number of detected peaks (secondary axes) vs. equilibration/sampling temperature (in °C). Figure S2: Distribution of the principal classes of volatile compounds detected. The following HS-SPME conditions have been used: t_eq_ = 20 min, t_sa_ = 35 min. The HS-SPME temperatures are reported into the brackets for each employed fiber. Table S1: HS-SPME/GC–MS results obtained using PDMS–DVB fiber (t_eq_ = 20 min and t_sa_ = 15 min at T = 80 °C). Table S2: HS-SPME/GC–MS results obtained using CAR–PDMS fiber (t_eq_ = 20 min and t_sa_ = 15 min at T = 80 °C).
