# Supplementary material for: Protective Effects Induced by a Hydroalcoholic Allium sativum Extract in Isolated Mouse Heart
Source: Nutrients. 2021 Jul 8;13(7):2332. doi: 10.3390/nu13072332 (PMC8308751; doi:10.3390/nu13072332)
Supplement: Supplementary file 1 [file nutrients-13-02332-s001.zip › nutrients-1255289-supplementary.pdf]

Protective effects induced by a hydroalcoholic *Allium sativum* extract in isolated mouse heart

Lucia Recinella[1], Annalisa Chiavaroli[1], Fabrizio Masciulli[1], Caterina Fraschetti[2], Antonello Filippi[2], Stefania Cesa[2], Francesco Cairone[2], Era Gorica[3], Marinella De Leo[3,4,5], Alessandra Braca[3,4,5], Alma Martelli[3,4,6], Vincenzo Calderone[3,4,6], Giustino Orlando[1], Claudio Ferrante[1], Luigi Menghini[1], Simonetta Cristina Di Simone[1], Serena Veschi[1], Alessandro Cama[1], Luigi Brunetti[1,*] and Sheila Leone[1,*]

1 Department of Pharmacy, G. d'Annunzio University of Chieti-Pescara, 66013, Chieti, Italy; lucia.recinella@unich.it (L.R.); annalisa.chiavaroli@unich.it (A.C.); fabriziomasciulli92@gmail.com (F.M.); giustino.orlando@unich.it (G.O.); claudio.ferrante@unich.it (C.F.); luigi.menghini@unich.it (L.M.); disimonesimonetta@gmail.com (S.C.D.S.); veschi@unich.it (S.V.); alessandro.cama@unich.it (A.C.); luigi.brunetti@unich.it (L.B.); sheila.leone@unich.it (S.L.).

2 Department of Drug Chemistry and Technology, Sapienza University of Rome, 00185, Rome, Italy; caterina.fraschetti@uniroma1.it (C.F.); antonello.filippi@uniroma1.it (A.F.); stefania.cesa@uniroma1.it (S.C.); francesco.cairone@uniroma1.it (F.C.).

3 Department of Pharmacy, University of Pisa, 56126, Pisa, Italy; era.gorica@phd.unipi.it (E.G.); marinella.deleo@unipi.it (M.D.L.); alessandra.braca@unipi.it (A.B.); alma.martelli@unipi.it (A.M.); vincenzo.calderone@unipi.it (V.C.).

4 Interdepartmental Research Center "Nutrafood: Nutraceutica e Alimentazione per la Salute", University of Pisa, Italy; marinella.deleo@unipi.it (M.D.L.); alessandra.braca@unipi.it (A.B.); alma.martelli@unipi.it (A.M.); vincenzo.calderone@unipi.it (V.C.).

5 CISUP, Centre for Instrumentation Sharing of Pisa University, 56126, Pisa, Italy; marinella.deleo@unipi.it (M.D.L.); alessandra.braca@unipi.it (A.B.).

6 Interdepartmental Research Center "Biology and Pathology of Ageing", University of Pisa, Italy; alma.martelli@unipi.it (A.M.); vincenzo.calderone@unipi.it (V.C.).

* Correspondence: luigi.brunetti@unich.it; Tel.: +39-0871-3554758; sheila.leone@unich.it; Tel.: +39-0871-3554658.

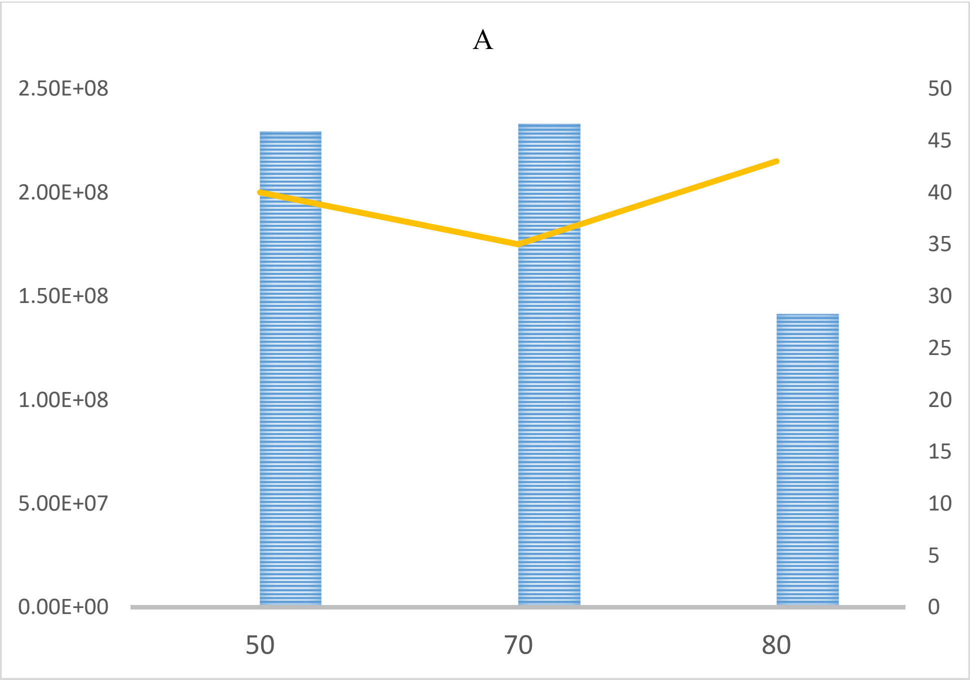

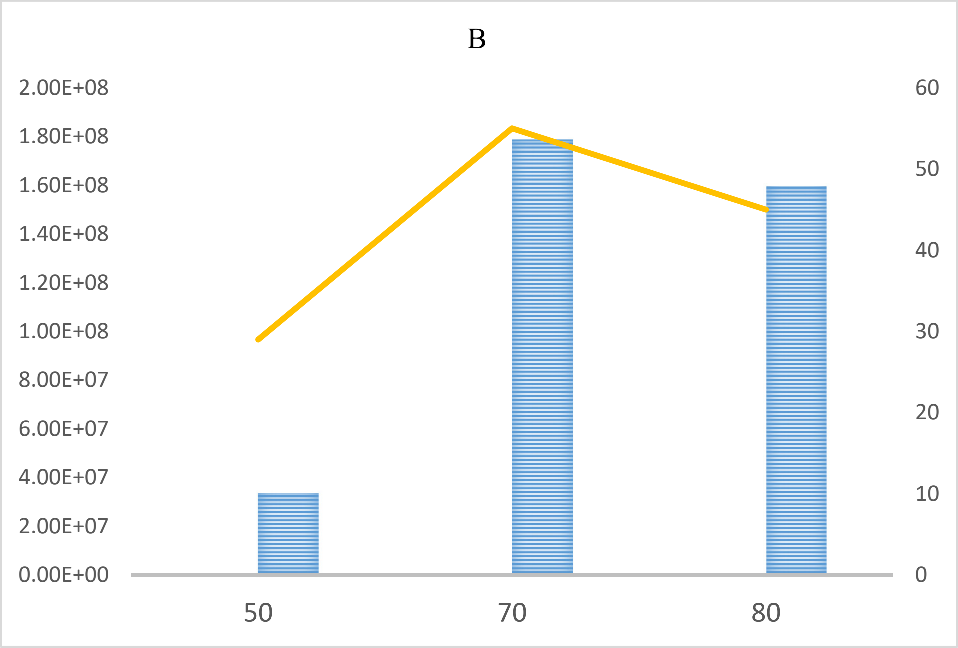

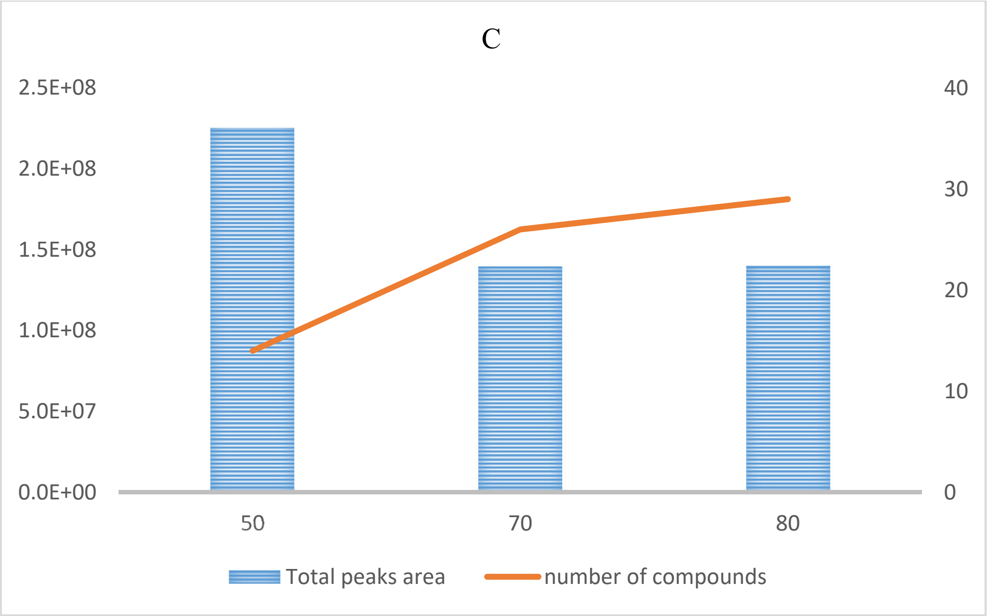

Figure S1. Optimization of HS-SPME conditions using A) DVB-CAR-PDMS, B) PDMS-DVB, C) CAR-PDMS fibers with the following settings: t_{eq} = 20 min, t_{sa} = 35 min. Total peaks area (primary axes) and number of detected peaks (secondary axes) vs equilibration/sampling temperature temperature (in °C).

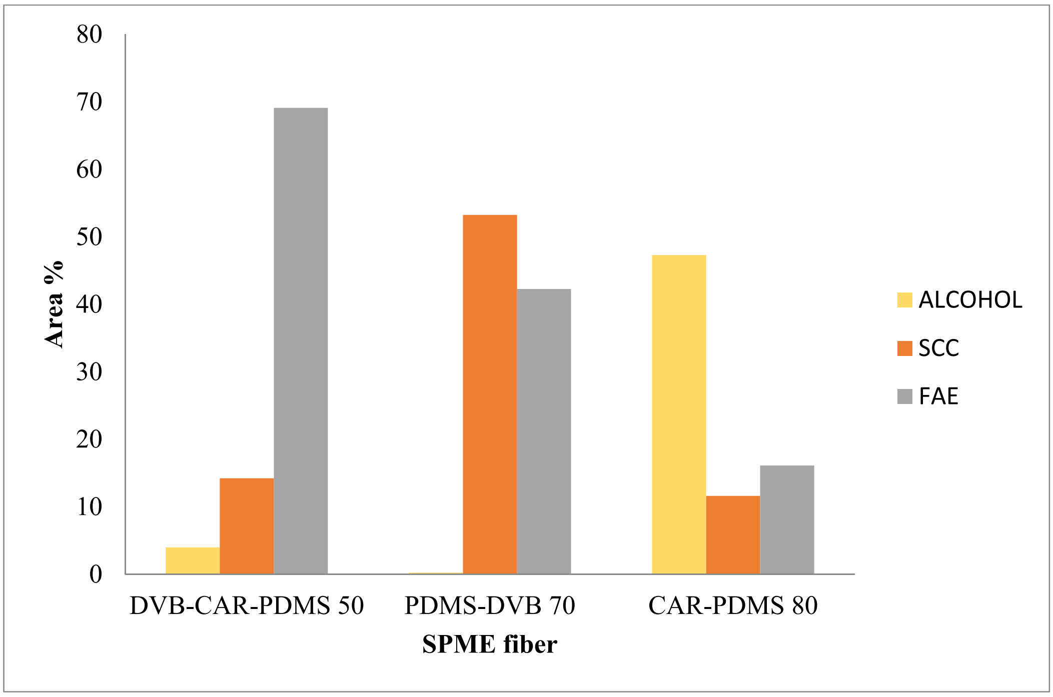

Figure S2. Distribution of the principal classes of volatile compounds detected. The following HS-SPME conditions have been used: t_{eq} = 20 min, t_{sa} = 35 min. The HS-SPME temperatures are reported into the brackets for each employed fiber.

Table S1. HS-SPME-GC-MS results obtained using PDMS-DVB fiber (t_{eq} = 20 min and t_{sa} = 15 min at T = 80 °C).

Compound	Class	Area%	RI	RI_L[a]
Propene[b]	Other	0,33	-	-
Allyl alcohol[b]	Alcohol	0,27	-	-
Trichloromethane[b]	Other	0,34	-	-
1,3 Propanedithiol	SCC	0,17	844	861
Diallyl sulfide	SCC	0,06	861	857
Methyl allyl disulfide	SCC	0,06	920	919
2,2,4,6,6-Pentamethyl heptane	Other	0,03	989	995
Diallyl disulphide	SCC	12,50	1083	1090
Allyl propyl disulfide	SCC	0,49	1097	1097
Ligustrazin	Other	0,26	1100	1083
Allyl methyl trisulfide	SCC	0,91	1142	1137
2,3,5-Trimethyl-6-ethyl pyrazine	Other	0,46	1172	1163
3-Vinyl 1,2 dithiacyclohex-5-ene	SCC	0,29	1192	1214
3-Vinyl 1,2 dithiacyclohex-4-ene	SCC	0,82	1219	1185
Diallyl trisulfide (allitridin)	SCC	37,52	1307	1300
Propenil propyl trisulfide	SCC	0,50	1319	1314
Eugenol	Alcohol	1,48	1368	1357
Allyl-1-methylthio propyl disulfide	SCC	0,25	1388	1387
Cyclopentane nonyl	Other	0,22	1450	1451
Diallyl tetrasulfide	SCC	3,23	1550	1539
2,4-Dimethyl-5,6-dithia-2,7-nonadienal	SCC	3,57	1776	1788
2,4-Dimethyl-5,6-dithia-2,7-nonadienal (isomer)	SCC	1,09	1792	1788
Myristic acid	Other	0,24	1798	1794
FAE (unidentified)	FAE	0,40	1827	-
Methyl hexadecanoate	FAE	0,82	1929	1925
Dibutyl phtalate	Other	0,22	1973	1960
Ethyl palmitoleate	FAE	0,33	1976	1975
Ethyl palmitate	FAE	6,88	1998	1993
Allyl stearate[b]	FAE	0,12	2093	-
Methyl linoleate	FAE	0,71	2100	2093
Methyl elaidate	FAE	0,15	2105	2107
Ethyl linoleate	FAE	8,70	2163	2164
Ethyl oleate	FAE	1,52	2168	2173
Ethyl linolenate	FAE	0,51	2171	2153
Methyl-9,12-heptadecadienoate[b]	FAE	0,99	2245	-
1-Hexyl-2-nitro cyclohexane[b]	Other	0,25	2250	-
13 unknown		13.31		
**Class**	**Area%**			
Alcohol	1.75			
SCC	61.46			
FAE	21.13			
Others	2.35			

[a]RI reported in literature [b]MS-only identification method.

Table S2. HS-SPME-GC-MS results obtained using CAR-PDMS fiber (t_{eq} = 20 min and t_{sa} = 15 min at T = 80 °C).

**Compound**	**Class**	**Area%**	**RI**	**RI_L[a]**
Allyl alcohol[b]	Alcohol	31.04	-	-
Acetic acid[b]	Other	8.38	-	-
Trichloromethane[b]	Other	3.80	-	-
2,3-Butanediol	Alcohol	28.06	800	788
Diallyl disulphide[b]	SCC	5.60	1084	-
Diallyl trisulfide (allitridin)	SCC	2.99	1308	1300
2,4 Dimethyl 5,6 dithia 2,7 nonadienal	SCC	2.03	1779	1788
2,4 Dimethyl 5,6 dithia 2,7 nonadienal (isomer)	SCC	0.92	1795	1788
Ethyl palmitate	FAE	7.41	2000	1993
Ethyl linoleate	FAE	4.88	2166	2164
Ethyl oleate	FAE	1.71	2171	2173
3 unknown		3.18		

**Class**	**Area%**
Alcohol	59.10
SCC	11.54
FAE	14.0
Others	12.18

[a]RI reported in literature [b]MS-only identification method.
